# Entrapped in the Cocoon: A Rare Case of Sclerosing Encapsulating Peritonitis in a Patient on Dialysis

**DOI:** 10.7759/cureus.96918

**Published:** 2025-11-15

**Authors:** Ritu Yadav, Lindsay Brown, Hamza Khan, Naman Suroya, Wina Yousman, Wei Xiang Wong

**Affiliations:** 1 Internal Medicine, Midwestern University Arizona College of Osteopathic Medicine, Cottonwood, USA; 2 Nephrology, University of Arizona College of Medicine - Tucson, Tucson, USA; 3 Nephrology, Verde Valley Medical Center, Cottonwood, USA

**Keywords:** abdominal cocoon syndrome, encapsulating sclerosing peritonitis, end stage renal disease (esrd), peritoneal dialysis (pd), tunneled dialysis catheter

## Abstract

Sclerosing encapsulating peritonitis (SEP), also known as abdominal cocoon syndrome, is a rare and potentially fatal condition characterized by the encasement of the small intestine within a dense fibrotic membrane. Fewer than 250 cases have been reported worldwide. We present a case of SEP in a patient on chronic peritoneal dialysis (PD).

A 66-year-old female with end-stage renal disease on chronic PD presented with worsening abdominal pain, distension, and hypotension. Laboratory evaluation revealed leukocytosis and an elevated peritoneal fluid white blood cell count. Peritoneal fluid cultures grew *Candida glabrata*, and treatment with intravenous antifungal and broad-spectrum antibiotics was initiated. Despite appropriate therapy and PD catheter removal, her symptoms persisted. Computed tomography (CT) of the abdomen and pelvis demonstrated loculated ascites and bowel loops encased in a fibrotic membrane, consistent with SEP. She was subsequently transferred to a tertiary care center for surgical decortication.

SEP is a rare but serious complication in patients on PD, often presenting as refractory peritonitis. Early diagnosis through clinical suspicion and imaging is crucial, as timely surgical intervention remains the definitive treatment to prevent bowel obstruction and improve outcomes.

## Introduction

Sclerosing encapsulating peritonitis (SEP) is a rare condition characterized by chronic, recurrent, or persistent intestinal obstruction due to diffuse adhesions and fibrosis of the peritoneum [1]. While idiopathic cases have been reported across diverse patient populations, SEP most commonly arises as a complication of peritoneal dialysis (PD), with its incidence increasing with longer duration of PD [2]. Fewer than 250 cases have been reported worldwide, with an estimated incidence ranging from 0.5% to 7.3%, particularly in patients undergoing prolonged PD (>5 years) [3]. The condition affects patients of all ages, including children as young as 5 years old, following extended PD use [4]. However, SEP appears to be more prevalent in tropical and subtropical regions, with a slight male predominance [5,6]. Additionally, younger PD patients are at higher risk, with an adjusted odds ratio of 0.81 per decade decrease in age [5]. Other risk factors for PD-associated SEP include prior episodes of peritonitis, glucose-based dialysates, prior abdominal surgery, hemoperitoneum, and PD withdrawal. Secondary causes of SEP include abdominal tuberculosis, systemic lupus erythematosus, chemotherapy, beta-blocker treatment, and ovarian disorders such as dermoid cyst rupture or luteinized ovarian thecomas [1].

Diagnosing SEP can be challenging due to its nonspecific and often progressive symptoms, including abdominal pain, distention, nausea, vomiting, early satiety, and unintentional weight loss. Patients on PD may also report changes in dialysate drainage, such as reduced ultrafiltration, blood-tinged fluid, or difficulty draining [1,4,5]. As the condition progresses, recurrent bowel obstructions often develop, leading to hospitalizations [5]. A thorough medical history and identification of relevant risk factors should prompt further diagnostic evaluation, including abdominal X-rays, barium studies, ultrasonography, and abdominal computed tomography (CT) [6]. Imaging findings in SEP commonly demonstrate dilated small intestinal loops with air-fluid levels and peritoneal calcification, while barium studies may reveal characteristic features, such as the “cauliflower sign” or “accordion pattern,” indicating central clustering of the small bowel loops [7,8]. Contrast-enhanced CT remains the most reliable diagnostic tool, typically demonstrating central accumulation of the small intestine encased by a dense membrane, with a contrast-free periphery - a hallmark of SEP [8].

SEP carries significant morbidity and mortality, particularly when intestinal obstruction progresses to ischemia, infection, systemic inflammatory response syndrome (SIRS), sepsis, or death [3,5,8]. Early recognition and prompt diagnosis are therefore critical to improving clinical outcomes [2]. This case study presents the clinical course, diagnostic challenges, and therapeutic management of SEP in a 66-year-old woman with cirrhosis and end-stage renal disease (ESRD) receiving PD.

## Case presentation

A 66-year-old female with a history of alcoholic cirrhosis, end-stage renal disease (ESRD) secondary to immunoglobulin A (IgA) nephropathy on chronic peritoneal dialysis (PD), hypertension, and diabetes presented with a 3-day history of progressive abdominal pain, distension, and fatigue. Two weeks prior, she reported that her PD catheter had partially dislodged due to accidental pull, but spontaneously repositioned. She applied over-the-counter topical antibiotic ointment and noted no redness, swelling, or discharge at the catheter site.

On presentation, she was hypotensive with a blood pressure of 85/53 mmHg, tachycardic, and exhibited abdominal distension with tenderness. The patient's blood pressure improved with intravenous fluids. Laboratory evaluation revealed a leukocyte count of 23,400/μL (4000-11000 /μL), lactic acid of 2.8 mmol/L (0.5-2.2 mmol/L), and a peritoneal fluid white blood cell count of 7,000/μL (normal <500/μL). Peritoneal fluid cultures grew *Candida glabrata*. Empiric intravenous (IV) anidulafungin was initiated. Given persistent symptoms and concern for refractory PD peritonitis, her PD catheter was removed, and a tunneled hemodialysis catheter was placed. Infectious disease consultation recommended the addition of IV clindamycin and ceftazidime.

**Figure 1:**
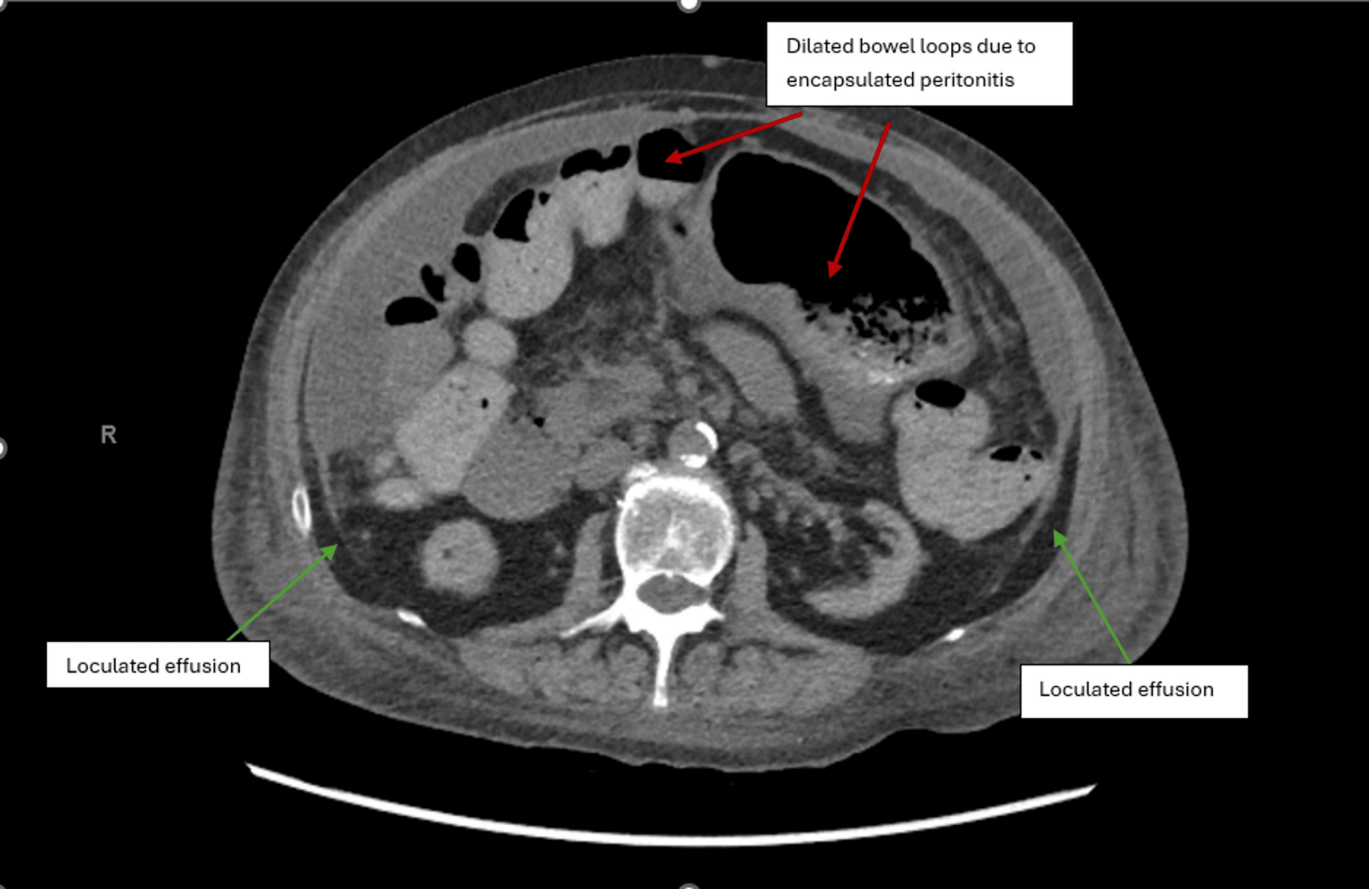
CT abdomen showing evidence of dilatation of the proximal small-bowel loops and loculated effusion resulting from thickening of the peritoneum

**Figure 2:**
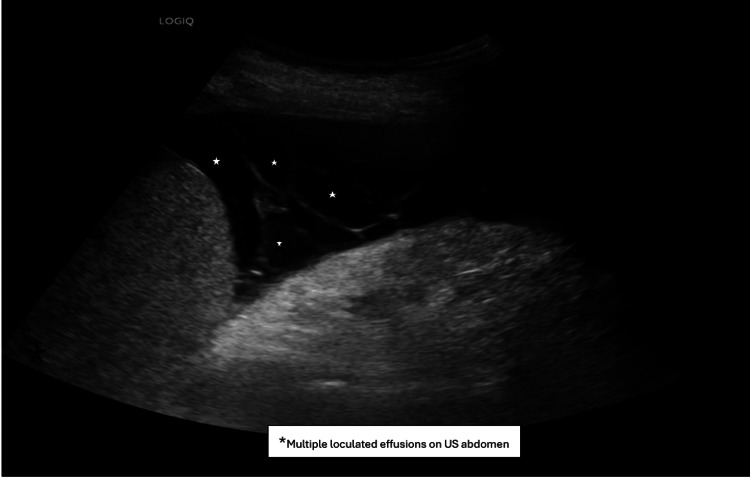
Ultrasound (US) abdomen showing loculated ascites

Despite appropriate antimicrobial therapy and source control, the patient’s abdominal pain and distension persisted. Computed tomography (CT) of the abdomen and pelvis demonstrated moderate, likely loculated, ascites, a right-sided pleural effusion, and peritoneal thickening with clustering of small bowel loops, raising suspicion for SEP, also known as abdominal cocoon syndrome (Figure 1). Ultrasound-guided paracentesis was performed by interventional radiology, which yielded an ascitic white cell count greater than 10,000/μL, further supporting the diagnosis (Figure 2). The patient’s condition necessitated transfer to a higher-level care center for definitive management. Surgical intervention, including decortication to remove the fibrotic cocoon encasing the bowel, was planned.

The patient was transferred to a higher-level care center for definitive management, but on arrival, she remained hemodynamically unstable with persistent hypotension and required vasopressor support. General Surgery evaluated her but determined that she was a poor surgical candidate. She underwent serial paracenteses and placement of a peritoneal drain for symptomatic relief. Despite aggressive supportive care, including cautious volume resuscitation with albumin and ongoing hemodialysis, her clinical condition deteriorated. She developed refractory hypotension, was unable to tolerate intermittent hemodialysis, and required transition to continuous renal replacement therapy. During her hospital course, she experienced a prolonged cardiac arrest lasting approximately 7-8 minutes, necessitating emergent intubation. Despite maximal ventilatory and hemodynamic support, she remained profoundly hypoxic. Given her poor neurological prognosis and ongoing multi-organ failure, goals-of-care discussions were held with her family, who elected to withdraw life-sustaining interventions, and the patient subsequently passed away.

## Discussion

SEP is a rare but life-threatening complication of recurrent PD-related peritonitis [1]. Although the exact pathophysiology remains unclear, overexpression of proinflammatory cytokines, neoangiogenesis, profibrotic gene activation, and mesothelial-to-mesenchymal transition are key contributors to disease progression [9]. This chronic inflammatory state leads to fibrosis and the eventual formation of a membrane encasing the bowel [1,9]. SEP staging is based on disease extent: Type 1 involves partial intestinal encapsulation, Type 2 involves the entire small intestine, and Type 3 includes additional organs such as the cecum, appendix, ascending colon, and ovaries [6].

Compared to other PD-related complications, SEP is notably severe and unique. Peritonitis, a common PD complication, occurs at rates ranging from 0.06 to 1.66 episodes per patient per year and is typically managed with intraperitoneal antibiotics and catheter removal in refractory cases [8]. However, recurrent or severe peritonitis, particularly due to *Staphylococcus aureus* or fungal infections, is a recognized risk factor for SEP [10]. Mechanical catheter-related complications, whether due to infection or displacement, may create a nidus for infection, increasing the risk of SEP [3,5]. In our patient, catheter dislodgement followed by spontaneous repositioning likely contributed. PD-related hernias, while not directly causing SEP, can place mechanical stress on the peritoneal membrane, potentially worsening the inflammatory and fibrotic processes that contribute to disease progression [11].

Diagnosis of SEP relies on clinical suspicion supported by imaging studies. While abdominal X-rays are often nonspecific, they may show features of small bowel obstruction, which can have a broad differential, including adhesions, hernias, malignancy, volvulus, intussusception, strictures, and foreign bodies [1,8]. Barium studies can be helpful in select cases to delineate bowel loops but are generally contraindicated in severe obstruction due to the risk of perforation and worsening obstruction [8,12]. Characteristic findings include conglomerated small bowel loops, the “cauliflower sign,” or an “accordion pattern” [13]. However, barium studies can be difficult to interpret due to large-volume ascites, common in patients with SEP. Ultrasound and CT imaging are more diagnostic, with loculated ascites being a hallmark finding in our patient. Ultrasound may also reveal the trilaminar sign, consisting of a superficial hyperechoic peritoneal membrane, a middle hypoechoic bowel wall, and a deep hyperechoic layer from bowel gas [14]. CT is the most sensitive modality, outlining the extent of encapsulation, peritoneal thickening, and features such as bowel angulation, kinking, and tethering, which indicate greater adhesion and surgical complexity. Calcifications and fibrosis, predictors of intraoperative complications, such as bowel gangrene and perforation, may also be seen [8,14]. In our patient, CT revealed loculated ascites, clustered bowel loops, and peritoneal thickening, confirming SEP.

The management of SEP is complex and often requires surgical intervention; however, initial therapy typically involves discontinuation of peritoneal dialysis, with mild cases managed conservatively through nasogastric decompression, bowel rest, and fluid resuscitation [6]. However, due to late-stage diagnosis in most cases, conservative therapy is frequently insufficient. Medical management with corticosteroids, tamoxifen, or colchicine aims to reduce inflammation and fibrosis but is rarely curative [15]. Advanced cases require surgical excision of the fibrotic membrane (enterolysis), which remains the definitive treatment [16,17]. In our patient, she was deemed a poor surgical candidate due to hemodynamic instability and multi-organ failure, so surgical intervention was deferred. She received supportive care, including serial paracenteses, peritoneal drainage, vasopressor support, and continuous renal replacement therapy. Despite these measures, her condition continued to deteriorate, ultimately leading to multi-organ failure and death.

Untreated SEP leads to rapid deterioration, culminating in complete mechanical obstruction [15]. Progressive bowel ischemia and necrosis may result in perforation, leading to bacterial peritonitis, sepsis, and multi-organ failure [18]. Given this rapid cascade, SEP carries a high mortality rate, with reported overall mortality between 25% and 55% [15]. In severe cases, mortality can escalate to 74% [14]. Intraoperative complications, including bowel perforation and gangrene, further increase these rates, often necessitating bowel resection [15,19].

Prevention of SEP relies on key strategies, including minimizing PD exposure in high-risk patients, utilizing alternative dialysis solutions, preserving kidney function, and ensuring early detection [3]. Because long-term PD (greater PD “vintage”) is a strong and repeatedly reported risk factor for SEP, consideration of transitioning high-risk patients off PD, either to hemodialysis or to kidney transplantation when feasible, is commonly recommended to reduce progression risk [20]. In patients continuing PD, glucose-free or low-glucose biocompatible solutions (e.g., icodextrin or amino acid-based solutions) help preserve peritoneal membrane function [21,22]. Preservation of residual kidney function through euvolemia maintenance, sodium restriction, diuretics, and avoidance of nephrotoxic agents further mitigates SEP risk [23]. Additionally, rigorous infection-prevention practices, including standardized exit-site and catheter care, prophylactic measures (e.g., nasal decolonization when indicated), and regular retraining/technique checks, reduce peritonitis rates and therefore lower one of the most important modifiable SEP risk factors [24,25].

Our case underscores the rapid and devastating course SEP can take, particularly in patients with prolonged PD and recurrent peritonitis. Despite early recognition and supportive measures, the patient’s advanced disease and critical condition precluded surgical intervention, highlighting the importance of prevention, early diagnosis, and timely escalation of care.

## Conclusions

SEP is a rare yet serious complication of PD that presents significant diagnostic and therapeutic challenges. This case underscores the importance of maintaining clinical vigilance in patients with prolonged PD use who exhibit progressive abdominal symptoms. Due to its nonspecific presentation and high morbidity, early diagnosis is essential for prompt intervention, which often requires surgical management. This case emphasizes the need for heightened awareness among clinicians caring for PD patients, as timely recognition and appropriate treatment can substantially improve patient outcomes and mitigate the life-threatening risks associated with SEP.
